# A trilogy of the oculomotor system: Part I: Ocular movements, finalities, and measurements

**DOI:** 10.5935/0004-2749.2024-0194

**Published:** 2024-11-11

**Authors:** Harley E. A. Bicas

**Affiliations:** 1 Department of Ophthalmology, Faculdade de Medicina de Ribeirão Preto, Universidade de São Paulo, Ribeirão Preto, SP, Brazil

**Keywords:** Angular measurement unity, eye movements, eye position measurement, eye position measurement accuracy, Fick’s system, Helmholtz’s system, ocular rotation, primary position of gaze, prism-diopter referential systems, superimposition of prisms

## Abstract

The paper starts discussing the teleological concept that eye motions - rotations and translations - serve to vision (which supports the notion that torsions are not voluntarily driven, since they do not contribute to expand the visual exploration of space). It proposes that the primary position of the eye (not “of gaze”) , the standard condition to measure them, must be defined as the coincidence of the orbital (fixed) and the ocular (movable) system of coordinates. However this becomes only a theoretic concept, since practical operations to obtain it are almost unfeasible. Besides, even a “simple” horizontal or vertical ocular rotation, though always occurring around a (presumably) fixed point (the center of ocular rotation) may be defined by different trajectories and magnitudes, depending on the two systems of measurement of eye positions and motions. Hence, in a graphical (plane) representation of such spherical coordinates, the so-called “tangent screen”, an ocular “tertiary” position - a combination of a horizontal and a vertical rotations - may be described by four different points. Or, conversely, a specific eye position may be defined by four sets of angular coordinates. The mathematical representation of variation of three special coordinates in a specific rotation is best made by a matrix disposition, so that, multiplication (not commutative) of three matrices (one for each specific plane) generates six different systems (permutations) of measurements. So, though , actually, there are multiple trajectories possible between two points in space, the *order* in which rotations are considered influences the final result. With different systems of coordinates for each rotation and different possible orders by which they may be considered, one reaches 48 alternative systems for their measurements. Unfortunately, up to now, there I is no an established convention to express ocular rotations. So, usually, people consider that a vertical prism superimposed to a frontally placed horizontal prism, or vice-versa, correspond to equivalent processes. The paper finishes discussing inconveniences of the clinically used unity to measure eye rotations (the prism-diopter) and proposes other unities as alternative solutions.

## Purpose of eye motions

Vision is the process by which stimulation of the eye by light reflected from objects in space is operated by the brain’s perceptual apparatus to create a holistic mental representation of the dimensions, relative positions and other physical qualities of those objects. The initial collection of visual data is the task of an assembled array of photoreceptors that are stimulated by the aforementioned reflected light. However, for photoreceptors to gather this information in a meaningful form, additional complementary factors are needed.

**Figure 1:**
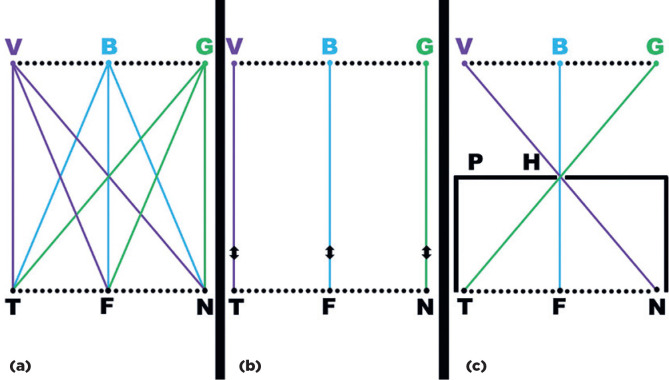
Representation of possible interactions between spatial stimuli and photoreceptors. The colored points labeled V, B and G represent points in space, while the black points T, F, and N represent photoreceptors in an arranged array. (a) With no selective process for specific relationships, each photoreceptor would receive information from all objects within visual range, preventing individuation of each stimulus. (b) Arranged couplings between each photoreceptor and a single spatial coordinate. (c) A small hole (H) in an opaque plate (P) interposed between an object in space and the eye limits the stimuli that reach the photoreceptor and facilitates a directional relationship between the photoreceptor and a specific spatial point.

Since the nature of the stimuli (light from a primary source - an emitter - or a secondary one, a reflector) is to propagate in all directions from its origin, a single photoreceptor should be able to receive such information from any object in visual range (Figure 1a). However, its simultaneous stimulation by the light from many objects impedes the discriminative individuation of each. This prevents it, and its fellow photoreceptors, from recording environmental stimuli in a manner that can be perceptually interpreted by the brain. To prevent this information overload, a specific correlation is established between each photoreceptor and a specific spatial area. The coupling of a photoreceptor with a single coor-dinate excludes any stimulation of that photoreceptor by light from any other point in space (*) (Figure 1b an 1c). Thus, the first complementary factor required for photoreceptors to accurately gather meaningful visual information is a limitation on the stimuli to which each photoreceptor is exposed. Such a limitation is achieved by the establishment of a specific directional correspondence between a photoreceptor and a spatial point in the visual field.

The second factor necessary for vision is the movement of the sensitive elements (photoreceptors) to avoid a visual field permanently limited to one portion of the environment. In principle, a specific visual direction is taken as a reference for the necessary movements. The line of sight that meets this criterion is the direction of the visual axis, central to the retina, that with the best visual discrimination, the so-called *primary* (or *principal*) *visual direction*, or *the visual axis.* Ocular motions are described as *rotations* when the visual axis is rotated around a reference point (the center of ocular rotations). They are described as *translations* when the position of the reference point is displaced. It can be seen from this that vision is dependent on motion.

## Reference systems for ocular movements

For mechanical studies, the eye may be considered a relatively rigid, spherical body, surrounded by elastic and viscous matter (muscle fibers, connective tissue, membranes, vitreous fluid, and ligaments). It is held in place by an almost hemispherical cup (the orbit of the skull) that allows the eye to make large, though partial, sliding rotatory movements. During those ocular rotations, the center of mass of the eye is slightly displaced from its original position, but such displacement, as other ocular translations relative to the orbit is small enough that it may be ignored for practical purposes. Thus, the eye may be considered to have a “fixed” center of ocular rotations with a constant position relative to the orbit. The orbital translations and rotations (produced by head movements) play an important role in the visual exploration of space. Therefore, two main referential systems for eye movements may be considered: that for movements of the eye itself (ocular rotations) and that for movements of the orbit (orbital rotations, or translations) with movements of the head.

Since the eye is fixed to the orbit, its geometric center (C) can be used as a fixed point in an orbital reference system. Similarly, the center of mass of the eye (the center of ocular rotations, R) can be used as a fixed reference point in an ocular reference system. In both systems, an imaginary line between the object of visual attention (O) and the fovea (F, sensorial center of the retina) is referred to as the visual axis as it is vital to vision. This line (OF) determines the effective gaze direction. Though points G and R do not necessarily coincide, they are too close that, for practical (clinical) grounds, they may be considered at the same place. Another ideal condition, though not always kept, is achieved when the visual axis coincides with the primary optical axis (an imaginary longitudinal line that passes the dioptric centers of the cornea and the crystalline lens in an anterior-to-posterior direction).

### A) The ocular reference system

**Figure 2:**
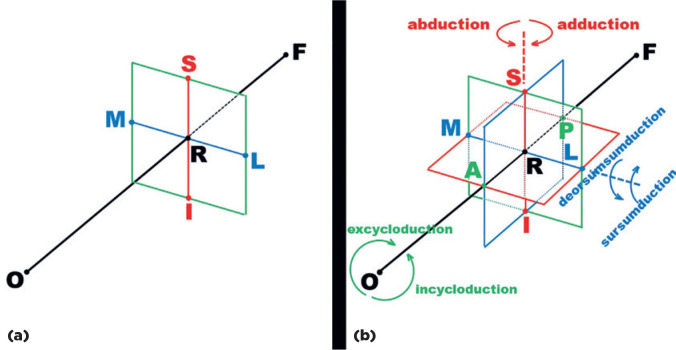
The ocular reference system of rotations of the left eye. (For the right eye, M and L change places.) (a) The visual axis (line ORF), between an object of visual attention (O) and the fovea (F) is perpendicular to an imaginary plane passing through reference point R. This reference point is the center of ocular rotations, around which they are measured. Two other mutually perpendicular axes (SI and LM) also pass though R; (b) The SI axis is perpendicular to the plane (in red) on which “horizontal” rotations are measured; the LM axis is perpendicular to the plane (in blue) on which “vertical” rotations are measured.

The visual (y-ocular, or AP-ocular) axis is perpendicular to an imaginary plane containing two other, mutually perpendicular axes, the x-ocular (or LM-ocular); and the z-ocular (or SI-ocular) axis, around which “vertical” and “horizontal” rotations respectively occur. Theses axes are on the vertical and horizontal planes, which exist in these spatial directions relative to the ground (Figure 2).

By convention, A, S and L in the ocular reference system (see Figure 2, for the left eye) are regarded as “directionally positive”. Hence from the perspective at each of those points, clockwise rotations around the respective axis are also seen as “positive”. Positive rotations around the vertical, SI-ocular axis are those moving in an A to M direction (adduction). Negative rotations around this axis are those that move in an A to L direction (abduction). Around the transverse, LM-ocular axis, positive rotations are those moving from A to S (sursumduction, or elevation), while negative rotations are those moving from A to I (deorsumduction or depression). Around the longitudinal, AP-ocular axis, positive rotations move from S to L (excycloduction or extorsion), while the counterclockwise (negative) rotations move from S to M (incycloduction or intorsion). Note that these movement descriptions only apply to the left eye. In the right eye, the relative positions of L and M are changed, but the rotation names remain the same (adduction from A to M, elevation from A to S and extorsion from S to L). The positive directions for adduction, elevation and extorsion are now counterclockwise. It is remarkable that cyclorotations (cyclotorsions) around the longitudinal axis (AP), tilt the visual image in a clockwise or a counterclockwise rotation, but do not produce any apparent enlargement of the visual space. This is most likely one of the reasons specific commands from the brain to produce such cyclotorsions are autonomic rather than consciously directed.

### B) The orbital reference system

A similar arrangement of mutually perpendicular axes (X, Y, and Z) with a single origin (C) is located on the orbit of the respective eye. The vertical (SI) orbital axis should coincide with the objective vertical line defined by the direction of the gravitational force at the place from where the measurement is considered. This line has to be used to define the objective horizontal plane, to which it is perpendicular. This means that the “true” vertical axis of each orbit are not rigorously parallel, however, the angular difference between them is so slight that it can be ignored for all “practical” purposes. Hence, a vertical plane containing the vertical axis and a horizontal plane perpendicular to the vertical axis can be defined. In practice, clinicians estimate the location of the vertical axis and/or the horizontal plane, by placing the head in a position that allows the vertical and/or horizontal lines of the human face to be so observed. Given the relative anatomical symmetry of the head and facial features and their approximate coincidence with the objective horizontal plane and the sagittal plane, the orbits will be positioned to allow estimation of the “objective” vertical axis (and/or horizontal plane) when the head is central and not tilted toward the right or to the left shoulders (an assumption of the required horizontal plane adjustment).

The common orthogonality of the three planes (and their respective axes) can now be used to define the frontal plane and/or horizontal lines (a transversal line contained by that plane, or a longitudinal one perpendicular to it). The “natural” (anatomical) orbital axis is a line that extends from the front to the apex of the orbit. However, it does not fulfill the criteria for what it is known as the progressive phylogenetical anteriority of the orbits, to support the concept of the human binocular vision - a fundamental requirement for the study of eye positions and movements - as the consequence of the superimposed visual fields. Therefore, the orbital frontal planes are conventionally defined by the (horizontal) LM-orbital axes, an imaginary transversal line from the right to the left sides of the head (so that both, the sagittal --- vertical) orbital planes can be considered parallel. When the subject is facing front and not turned to the right or left, one can infer the correct adjustment of the frontal plane of the orbits.

Once these orbital objective planes and their respective axes have been defined, they can be made coincident to the respective planes and axes of the eye, giving eye “positions” and rotations a fixed (to the head) and objective system of references for their measurements. The position of the visual axis at such a coincidence of the orbital and corresponding ocular axes is regarded as the primary position of the respective eye and the landmark used in measurements of (other) eye positions and movements.

### C) The primary position of the eye

The simplicity of the concept of a primary position of the eye as the complete coincidence of the respective axes of the eye and the orbit poses some disturbing comments:

1. Unfortunately, despite this quite simple theoretical definition of the primary position of the eye, the practical operations by which it may be obtained are complex and near impossible to work out. Furthermore, there is a lack of standards with which to objectify orbital planes (such as a plumb line to define the vertical and horizontal planes) and obstacles to applying guidelines in practice (facial asymmetries, though sometimes minute, are always present, and these interfere with the perpendicular relationship between the expected horizontal and vertical reference lines). This makes any preemptive affirmation that the head position is perfectly adjusted to an objective horizontal plane imprudent. Nonetheless, possible conventions have been proposed^(^1^)^. To summarize, approximate of the frontal plane of the head to an acceptably symmetrical position (from the perspective of the clinician) and of the sagittal plane of the head to ensure it is not inclined relative to the objective vertical line, can be clinically satisfactory. However, the lack of established standards for improved accuracy of such approximations warrants further discussion. The major barrier to the development of such standards is determining the correct adjustment of the horizontal plane of the head. An “erect” front-facing head position is not easy to achieve precisely. This is apparent when one attempts it in front of a mirror. The reader may wish to attempt this and try to identify the correct balance point (i.e., that corresponding to the objective, horizontal plane) among a relatively extended range of more “haughty” or “depressed” head positions.
2. The primary position of gaze has been proposed as a reference direction for the measurement of eye positions and movements. In such an instance, “gaze” is perhaps not the best descriptor. Gaze generally refers to direction of the visual axis (attentively or not) toward a particular spatial point. As the eye may be rotated around a given direction (as, for example, the direction of the visual axis), innumerable eye positions (torsions) are possible-with-the-same direction of gaze. Rigorous measurements of ocular torsion cannot be guaranteed, since only the longitudinal axis can be defined using proper referential conditions. The other ocular axes (transverse and sagittal) cannot. Also, ocular torsion may be only measured using information given by the owner of the eye under evaluation. Consequently, a specific position of the eye cannot be determined using only the direction of the longitudinal (visual) axis.
3. Similarly, the adjustment of a pair of equivalent axes from each monocular system (for instance, the longitudinal, or sagittal axes of both eyes) is insufficient to define the primary position of the eye. The coincidence of the transverse (horizontal) axes of both eyes does not mean that the respective vertical axes are parallel.
4. The origin of the orbital axes (C) should not be taken to be the center of the orbit, nor the (geometrical) center of the eye. While it is not necessary for mea-surements of eye movements (among which ocular rotations prevail), it is more convenient C be made to coincide with R, the center of ocular rotations.
5. The permanent coincidence of the ocular and orbital axis, that is, the fixed position of the eye relative to the orbit is a theoretical simplification used in the study of ocular rotations. But it has previously been shown that such a conceptual point would be displaced (translated) during the rotations. At best, the visual axis (or “line of sight”) may rotate around a fixed point in space (orbit) without passing by R^(^2^,^3^)^.
6. Since the center of ocular rotation (R) and the geometric center of the eye (G) are not equivalent (Figure 3), points on the scleral surface (such as the insertion points of muscular fibers) are equidistant from G, but not from R. For instance, while the respective coordinates of M and L have the same distance to G and maintain this distance during eye rotations, they have different values when considered relatively to R (Figure 3). This is because, even in a perfectly spherical eye, the rotational arms of the insertions for each muscle fiber (or the rotational “radius of the eye”) are unequal.
  **Figure 3:**
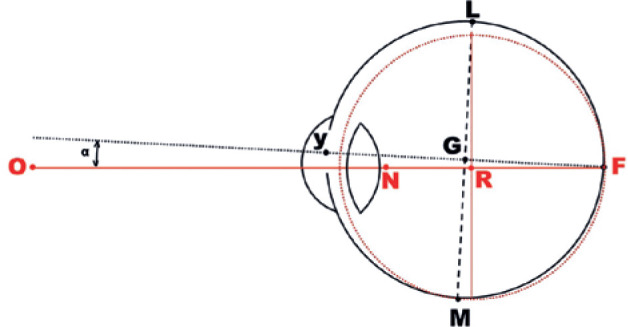
Schematic representation of the visual (unbroken red line, ONRF) and geometric (dotted black line, yGF) axes of the right eye (upper view). The visual axis is the straight line between the object of visual attention (O) and the fovea (F). Ideally, it passes through the center of ocular rotations (R) and is coincident to the optical axis of the dioptric ocular system (which passes through the center of corneal curvature and the - supposedly centered - dioptric curvatures of the crystalline lens). If F is made coincident to the posterior pole of the eye, the geometric center of the eye (G) is usually slightly displaced toward the lateral (temporal) side and in front of R. The angle between the visual axis (ORF) and the geometric ocular axis (yGF) is called *alpha* (*).

*The terminology used to describe the ocular axes is used somewhat inconsistently. However, the visual axis is usually deemed to be the straight line between a spatial point and the center of the fovea. It has been proposed that the visual axis may have nodal points generated by the optical properties of the eye. These points would be contained by the optical axis, a line that passes through the corneal apex and the centers of curvature of the anterior and posterior diopters of the cornea an the crystalline lens. Ideally, the visual axis provides the best optical image, but this is not always the case if there is not the necessary alignment of the several centers of curvature. Instead of the visual axis, some authors refer to a line of vision (between a spatial point and the center of the pupil), a pupillary axis (perpendicular to the center of the cornea and the center of the pupil). Based on these different axes, theoretical angles have been proposed, including *kappa* (the angle between the visual and pupillary axes) and *lambda* (the angle between the pupillary axis and the line of vision), among others.

## Measurements of eye rotations

As a matter of simplification, the eye may be considered as a perfect sphere, which geometric center is the center of its rotation (C). All of its superficial points are, then, equidistant from C and rest over imaginary “large” circles (e.g., the equator or any of its perpendicular sections, the circles of longitudes). Except for the “poles” all other points of the ocular surface may be also defined as resting over imaginary “small” circles (as those of “latitudes”, that is, circles parallel to a considered equator). Therefore, similarly to any point of the terrestrial surface, superficial points of a sphere may have a coordinate of “longitude” and one of “latitude”. All the same, a sphere can be divided by curved (and parallel) lines of “small” circles (lines of “latitudes”) and be divided by lines of “large” circles (lines of longitudes), mutually perpendicular. For the case of the eye, although *all* ocular rotations occur, *always*, around C, the trajectory of different points of the ocular surface describe arcs of different magnitudes, all of them centered at a normal (perpendicular) axis to the plane of the respective rotation.

**Figure 4:**
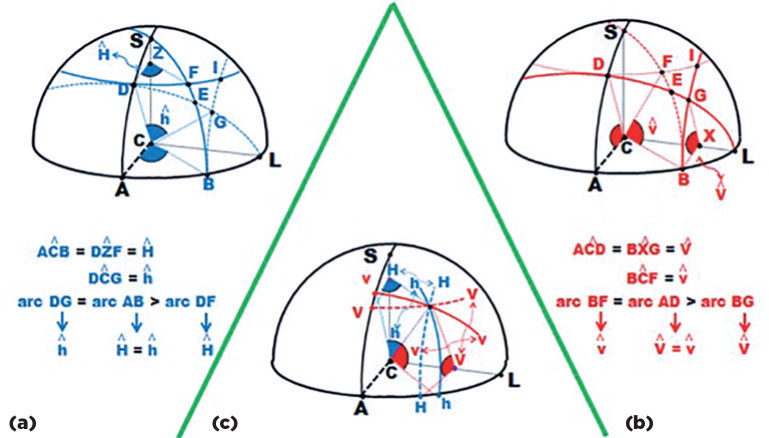
Representation of systems of angular coordinates in a spherical body. *Horizontal* angles, named as “h”, are taken relatively to C, in lines of large circles (ABL and DEGL) ; a specific angle (in different large circles) has arcs with the same length (e.g., AB and DG). When taken relatively to a point (Z) of the vertical axis (CS), in lines of small circles, parallel to the horizontal plane (DFI) they are named as “H”; for a specific angle (ACB = DZF) the length of arcs (AB and DF) are unequal. (b) *Vertical* angles, named as “v”, are taken relatively to C, in lines of large circles ADS and BEFS); a specific angle (in different large circles) has arcs with the same length (e.g., AD and BF). When taken relatively to a point (X) of the transversal axis (CL), in lines of small circles, parallel to the sagittal plane (BGI) they are named as “V”; for a specific angle (ACD = BXG) the length of arcs (AD and BG) are unequal. (c) A specific point of the surface (crossing of blue and red lines) may be defined by *any* among four combinations of those angular coordinates (H and V, H and v, h and V or h and v), with different magnitudes (h > H and v > V).

For instance, a measurement of a *horizontal* angle (“H”) may be measured around a fixed vertical axis (CS) so that arcs in different planes have *different* lengths (AB or DF, Figure 4a). But if the angles are measured relatively to the center of rotation (C), the rotational arcs have the same length AB and DG, Figure 4a).

All the same, the measurement of a *vertical* angle may be given by the same pair of conventions. If they are considered around a fixed transversal axis (CL, Figure 4b), the arcs have different lengths (AD and BG) but correspond to the same angle, defined as “V”. Arcs measured from C have the same length (BF and AD) and are defined as “v”. Angle measurements taken from the center of ocular rotations (“h” and “v”) are said to be of the *ocular* system of coordinates. Those measured from points of fixed axes in space (“H” and “V”) are said to be of the *orbital* system of coordinates.

Therefore, even if one consider only a “simple” rotation, taken from the primary position in a fundamental (horizontal or vertical) plane, a point may have two trajectories and two final positions. For instance, a vertical rotation of 30° for the point B is the arc BF if one considers “v”, but the arc BG if one considers “V”. All the same, a horizontal rotation of 30° for point D means arc DG if one considers “h”, or arc DF if one considers “H”.

When one consider the case of both, vertical and horizontal angles with the same value (say 30°), *four* diffe-rent combinations result (Figures 4a and 4b): “HV”, if both measurements are related to the orbital coordinates (point I); “Hv”, if the horizontal measurement is related to the orbital system and the vertical measurement is related to the ocular system of coordinates (point F), or vice-versa (“hV”, point G), or “hv” if both measurements are related to the ocular system of coordinates (point E). Conversely, since the same set of angular coordinates corresponds to four different positions of a point, the position of a point may correspond to four different sets of coordinates, that is, HV, Hv, hV or hv (Figure 4c).

If one considers the three (horizontal, vertical and torsional) rotations, the possible combinations become eight (HVT, HVt, HvT, Hvt, hVT, hVt, hvT, hvt).

- Consider Figure 4a as similar to the terrestrial system of coordinates, with line AB as the equator and AD as the meridian of a standard longitude. F is then defined by a longitude (the horizontal angle) ACB = DZF, and a latitude BCF (the vertical angle) = ACD. This system of coordinates for measurement of horizontal rotations (as “longitudes”) and vertical rotations (as “latitudes”) is that of Fick.
- The system whereby vertical rotations “are measured using the angle of elevation” and horizontal rotations “using the angle of Azimuth” (coordinates for point G, Figure 4b) is that of Helmholtz.

### Planar representation of spherical coordinates

**Figure 5:**
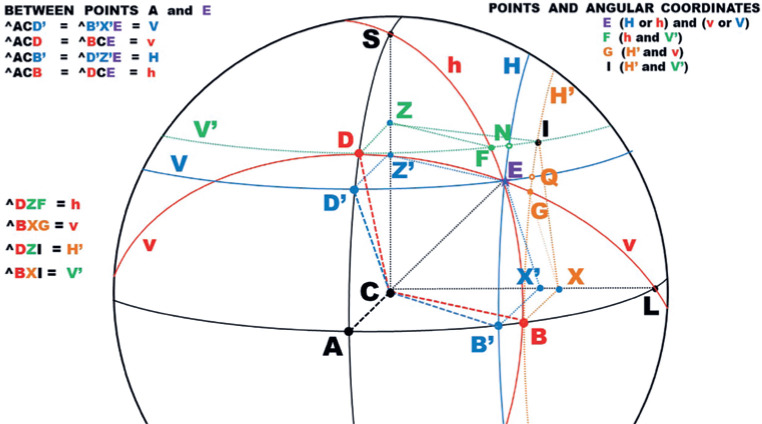
Schematic representation of the angular coordinates of superficial points of a spherical body. Points A, B, C, D, E, F, G, I, S and L have already been defined (see Figure 4). To define point E, two new lines are considered. These are the blue line D’E (elevation V), defining arc D’Z’E (which represents angle “H”), which is parallel to the horizontal plane; and the blue line B’E, defining arc B’X’E (which represents angle “V”), which is parallel to the sagittal plane. Point E may be defined by the angular coordinates H (arc D’Z’E), V (arc B’X’E), h (arc DCE), and v( arc BCE); that is, by four pairs of angular coordiantes (HV, hV, Hv, or hv). Note that, for the different coordinates of a specific point (e.g., E), v (= ACD = BCE) > V (=ACD’ = B’X’E) while h (= ACB = DCE) > H (= ACB’ = D’Z’E). Two other parallel lines are also considered, one for elevation V’ (the green line passing through D, F and I) and the other defining rotation H’ (the ochre line passing through B, G and I). This gives the systems of angular coordinates for points F (hV’), G (H’v) and I (H’V’). So, for the same coordinates of different points (E, F, G, I), BXG = v < BXI = V’; and DZF = h < DZI = H’.

**Figure 6:**
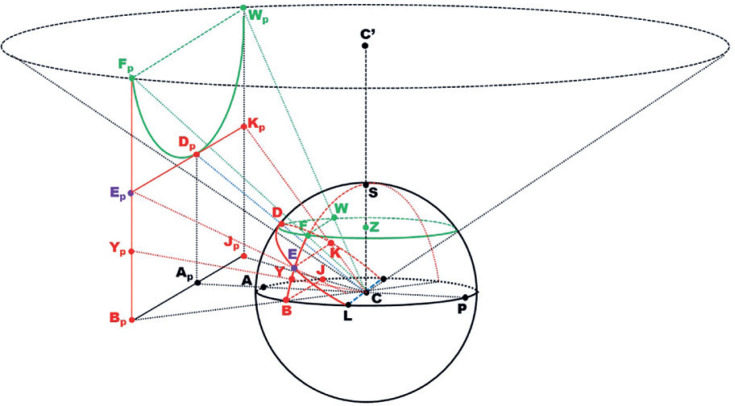
Projections of points on a spherical surface from its center (C) on a flat plane. The projection from C corresponds to an imaginary conical figure. Arcs of large (maximal) circles (e.g., AD, BYEF, BAJ, EDK) are represented by straight lines (respectively A_p_D_p_, B_p_Y_p_E_p_F_p_, B_p_A_p_J_p_, E_p_D_p_K_p_), while while arcs of small circles (e.g., FDW) are represented by arcs of hyperbolas. The flat plane is perpendicular to the plane to which the axis of the imaginary cone (CC’) is also perpendicular.

As shown in Figure 5, the spherical arcs and points of a sphere may be represented on a flat plane, perpendicular to one of its main axes, using their projections from the respective center of reference. In ocular rotation measurement, this would be the longitudinal axis (the visual axis) (Figure 3). The projections from this are called *central, polar, zenithal* or *gnomonic* projections. The flat plane, supposed to tangentially touch the anterior pole of the eye (A) is called a *tangent screen*. In the case depicted in Figure 6, the positions of the anterior pole of the sphere (A) and its representation on a flat plane (A’) are coincident and serve as the origin of a system of (planar) coordinates. In such a plane, the distance between any projected point (P_p_) and its origin (A_p_ = A), distance P_p_A_p_ (= P_p_A) is given by a straight line perpendicular to the line between this origin (A_p_) and the center of ocular rotations (C), which is the radius of curvature of the sphere (r). In other words, P_p_A_p_ is related to constant (r) by a simple tangent scale. For example, for point K (and its projection K_p_), A_p_K_p_ = r tan a = [(A_p_J_p_)^^2^^ + (J_p_K_p_)^^2^^]^½^.

As can be seen in Figure 6, even when the sphere does not touch the flat plane (screen), but is separated from it by distance AA_p_ = d, the tangent scale still holds: A_p_K_p_ = k tan a, where k = r + d. This is the reason for the use of the general term “*tangent screen*” in the figures showing measurements that use this type of gnomonic projection.

**Figure 7:**
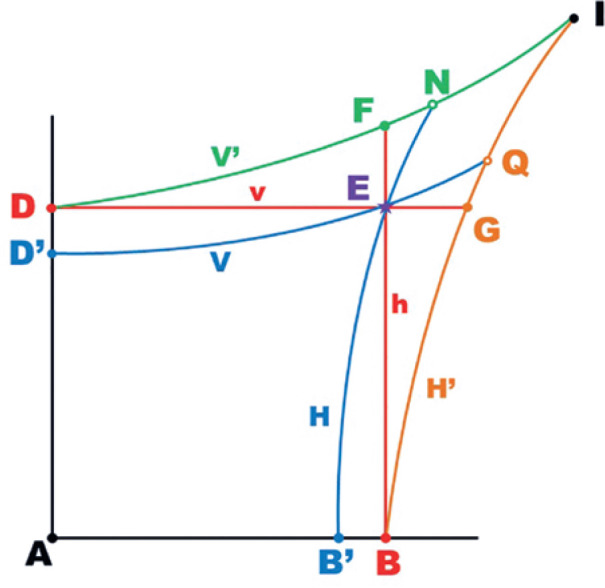
Graphical representation of the determination of angular coordiantes on a spherical body, using its gnomonic projection on a flat plane (the “*tangent screen*”). The straight (red) lines correspond to large circles of the sphere (angles measured from its center), while the curved (blue, green and ochre) lines correspond to small circles of the sphere (angles measured from the horizontal and vertical axes). Therefore, a single point (e.g., E) may have four different sets of angular coordinates, according to the combinations of the selected system of coordinates for horizontal (H or h) and vertical measurements ( V or v). Different points may be represented by the same Angular coordinate values but differ depending on the type of measurement made: F (h and V’), G (v and H’) and I (H’ and V’); or N (H and V’) and Q ( H’ and V). (Note that v = V’ for point D, while h = H’ for point B).

**Figure 8:**
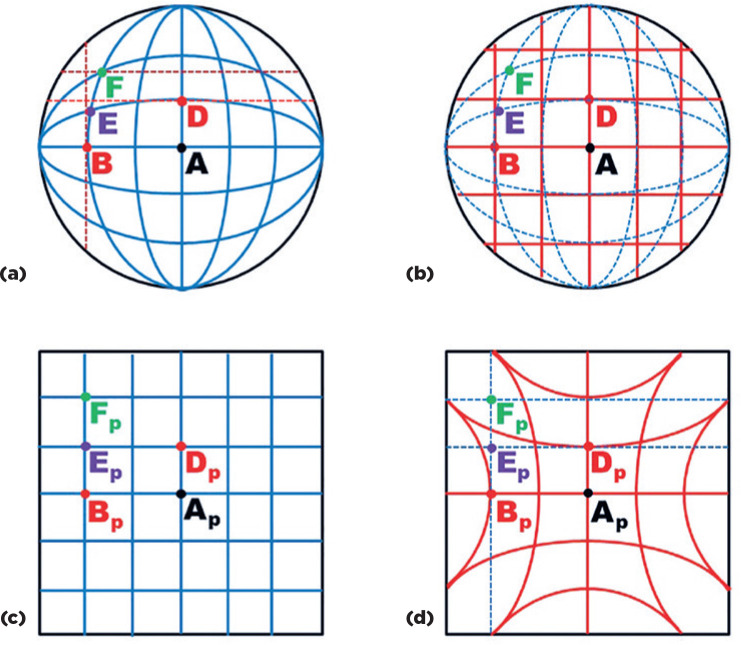
Graphical representation of the superficial lines of a spherical body taken as angular coordinates from points on the sphere: (a) Large circles, represented by straight (the horizontal and the vertical equators) and curved (equatorial sections of the sphere passing through its center) blue lines (front view); (b) Small circles, represented by red lines, parallel to the horizontal and vertical equators (front view); (c) and (d) The respective gnomonic projections on a flat plane. Large circles are represented by straight (blue) lines , while small circles are represented by hyperbolic (red) curves (back views).

Figure 7 provides a graphical representation of the points from Figure 5 that could be represented by the same set of angular coordinates on a flat plane of gnomonic projections. Note that, in a gnomonic projection, the curved lines of large circles (“the equator” and “meridians”) are represented by straight lines, while those of small circles (parallel to large circles) are represented by hyperbolic curves (Figure 8).

## Mathematical presentation of different systems of ocular rotations

We have now seen that although all ocular rotations (whether horizontal or vertical) are effectively centered at C (the center of ocular rotations), as “h” or “v”, the coordinate system used to determine the position of a point on the ocular surface may be defined by other referential concepts (as “H” or “V”). Using the different criteria of each system to calculate the coordinates of the point in question (e.g., point E in Figure 7), four combinatioons emerge (HV, Hv, hV and hv).

It has been also shown that betweeen two points (e.g. A and E in Figures 4 to 8), infinite paths are possible (for instance from A to D and D to E); or from A to B and B to E; or directly from A to E; or any other). That is, the coordinates of a point (E) are not dependent on the temporal order in which rotations are performed. Note, however, that for a given point (e.g., point E, as shown in Figures 5 and 7), H < h, and V < v, so that establishing a criterion for measurement of a horizontal rotation (H or h) influences the “subsequent” measurement of the vertical rotation (V or v), and vice-versa. For instance, if one defines a horizontal rotation of 30° as “h”, “H” will be < 30°.

**Figure 9:**
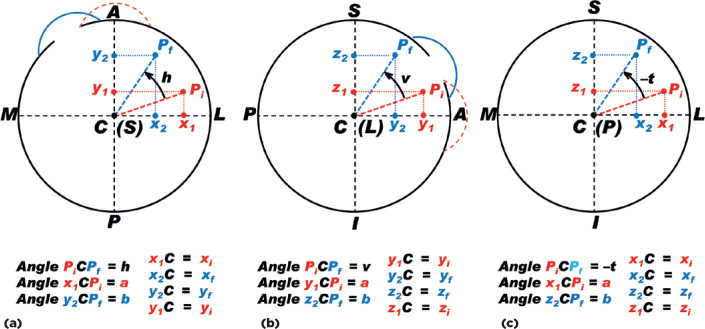
Schematic representation of the positions of a *superficial* point of the right eye, before P_i_ (x_i_, y_i_, z_i_) and after, P_f_ (x_f_, y_f_, z_f_) rotations of adduction (a), sursumduction (b) and intorsion (c). The views for the horizontal (a) and the sagittal (b) planes are from the “positive” poles (S and L) while that for the frontal (c) plane is from the posterior (“negative”) pole of the eye (P) for which the signal for torsion (t) must be negative.

Let us suppose that all rotations on different planes (Figure 9) are ascertained from the same center (C).

In Figure 9, a + b + # = 90° (where # represents h, v or -t) so that


$$\begin{array}{rl} & \sin(a+b)=\sin\left(90^{\circ }-h\right)=\cos\#=(\sin a)(\cos b)+(\cos a)(\sin b)\\ & \cos(a+b)=\cos\left(90^{\circ }-h\right)=\sin\#=(\cos a)(\cos b)-(\sin a)(\sin b) \end{array}$$

But in Figure 9a, CP_i_ = CP_f_ = k, so:


$$\sin a=y_{i}/k\;\cos a=x_{i}/k\;\sin b=x_{i}/k\;\cos b=y_{i}/k$$

Hence:


(F. I) $$\cos h=\left(y_{i}/k\right)\left(y_{f}/k\right)+\left(x_{i}/k\right)\left(x_{i}/k\right)=\left[\left(x_{i}\right)\left(x_{f}\right)+\left(y_{i}\right)\left(y_{f}\right)\right]/k^{2}$$

(F. II) $$\sin h=\left(x_{i}/k\right)\left(y_{f}/k\right)-\left(y_{i}/k\right)\left(x_{f}/k\right)=\left[\left(x_{i}\right)\left(y_{f}\right)-\left(y_{i}\right)\left(x_{i}\right)\right]/k^{2}$$

Isolating y_f_ from F. I and F. II:


$$\begin{array}{c} y_{f}=\left[k^{2}(\cosh)-\left(x_{i}\right)\left(x_{f}\right)\right]/y_{i}=\left[k^{2}(\sin h)+\left(y_{i}\right)\left(x_{i}\right)\right]/x_{i}\to \\ \left(x_{i}\right)\left[k^{2}(\cos h)-\left(x_{i}\right)\left(x_{f}\right)\right]=\left(y_{i}\right)\left[k^{2}(\sin h)+\left(y_{i}\right)\left(x_{f}\right)\right]\to \\ k^{2}\left[x_{i}(\cos h)-y_{i}(\sin h)\right]=x_{f}\left[{\left(y_{i}\right)}^{2}+{\left(x_{i}\right)}^{2}\right] \end{array}$$

However, (y_i_)^^2^^ + (x_i_)^^2^^ = (y_f_)^^2^^ + (x_f_)^^2^^ = k^^2^^, so that:


(F.III) $$x_{i}(\cos h)-y_{i}(\sin h)=x_{i}$$

Alternatively, isolation of x_f_ from F. I and F. II:


(F. IV) $$\begin{array}{rl} & x_{f}=\left[k^{2}(\cos h)-\left(y_{i}\right)\left(y_{i}\right)\right]/x_{i}=\left[\left(x_{i}\right)\left(y_{i}\right)-k^{2}(\sin h)\right]/y_{i}\to \\ & \left(y_{i}\right)\left[k^{2}(\cosh)-\left(y_{i}\right)\left(y_{j}\right)\right]=\left(x_{i}\right)\left[\left(x_{i}\right)\left(y_{j}\right)-k^{2}(\sinh)\right]\to \\ & k^{2}\left[y_{i}(\cosh h)+x_{i}(\sinh h)\right]=y_{i}L{\left(x_{i}\right)}^{2}+{\left(y_{i}\right)}^{2}]\to \\ & y_{i}(\cos h)+x_{i}(\sin h)=y_{f} \end{array}$$

Similarly, in Figure 9b (changing “h” to “v”, “x” to “y” and “y” to “z”), equations F.III and F. IV become:


(F.V) $$y_{t}(\cos v)-z_{t}(\sin v)=y_{f}$$

(F. VI) $$z_{t}(\cos v)+y_{t}(\sin v)=z_{f}$$

Finally, in Figure 9c, a + b = 90 - (- t), hence:


(F. VIII) $$\begin{array}{c} \sin(a+b)=\sin(90+t)=\cos t=(\sin a)(\cos b)+(\sin b)(\cos a)\to \\ \cos t=\left(z_{i}/k\right)\left(z_{f}/k\right)+\left(x_{f}/k\right)\left(x_{i}/k\right)=\left[\left(z_{i}\right)\left(z_{f}\right)+\left(x_{i}\right)\left(x_{f}\right)\right]/k^{2}(F.VIl)\\ \cos(a+b)=\cos(90+t)=-\sin t=(\cos a)(\cos b)-(\sin a)(\sin b)=\\ -\sin t=\left(x_{i}/k\right)\left(z_{f}/k\right)-\left(z_{i}/k\right)\left(x_{f}/k\right)\to \sin t=\left[\left(x_{f}\right)\left(z_{i}\right)-\left(x_{i}\right)\left(z_{f}\right)\right]/k^{2} \end{array}$$

By equalization of z_f_ in F. VII and F. VIII:


(F. IX) $$\begin{array}{c} \left[k^{2}(\cos t)-\left(x_{i}\right)\left(x_{f}\right)\right]/z_{i}=z_{f}=-\left[k^{2}(\sin t)-\left(x_{f}\right)\left(z_{i}\right)\right]/x_{i}\to \\ k^{2}(\cos t)x_{i}-{\left(x_{i}\right)}^{2}\left(x_{f}\right)=-k^{2}(\sin t)z_{i}+\left(x_{f}\right){\left(z_{i}\right)}^{2}\to \\ k^{2}\left[(\cos t)x_{i}+z_{i}(\sin t)\right]=\left(x_{f}\right)\left[{\left(z_{i}\right)}^{2}+{\left(x_{i}\right)}^{2}\right]\to \\ x_{f}=x_{i}\cos t+z_{t}\sin t \end{array}$$

And, by equalization of x_f_ in F. VII and F. VIII:


(F. X) $$\begin{array}{c} \left[k^{2}(\cos t)-\left(z_{p}\right)\left(z_{i}\right)\right]/\left(x_{i}\right)=x_{f}=\left[k^{2}(\sin t)+\left(x_{i}\right)\left(z_{f}\right)\right]/\left(z_{i}\right)]\to \\ k^{2}(\cos t)\left(z_{i}\right)-\left(z_{f}\right){\left(z_{i}\right)}^{2}=k^{2}(\sin t)\left(x_{i}\right)+\left(z_{f}\right){\left(x_{i}\right)}^{2}\to \\ k^{2}\left[(\cos t)\left(z_{i}\right)-(\sin t)\left(x_{i}\right)\right]=\left(z_{f}\right)\left[{\left(x_{i}\right)}^{2}+{\left(z_{i}\right)}^{2}\right]\to \\ z_{f}=-\left(x_{f}\right)\sin t+\left(z_{f}\right)\cos t \end{array}$$

Equations F. III and F. IV may be represented in matrix form:


(F. XI) $$\begin{array}{c} \left[\begin{array}{c} x_{i}(\cos h)-y_{i}(\sin h)+z_{i}(0)\\ x_{i}(\sin h)+y_{i}(\cos h)+z_{i}(0)\\ x_{i}(0)+y_{i}(0)+z_{i}(1) \end{array}\right]=\left[\begin{array}{l} x_{f}\\ y_{f}\\ z_{f} \end{array}\right]\to \left[\begin{array}{ccc} \cosh & -\sin h & 0\\ \sin h & \cosh & 0\\ 0 & 0 & 1 \end{array}\right]\left[\begin{array}{l} x_{i}\\ y_{i}\\ z_{i} \end{array}\right]=\left[\begin{array}{l} x_{f}\\ y_{f}\\ z_{f} \end{array}\right]\\ \to \left[\begin{array}{l} M_{h} \end{array}\right]\cdot \left[\begin{array}{l} x_{i}\\ y_{i}\\ z_{i} \end{array}\right]=\left[\begin{array}{l} x_{f}\\ y_{f}\\ z_{f} \end{array}\right] \end{array}$$

As can equations F. V and F. VI:


$$\left[\begin{array}{c} x_{i}(1)+y_{i}(0)+z_{i}(0)\\ x_{i}(0)+y_{i}(\cos v)-z_{i}(\sin v)\\ x_{i}(0)+y_{i}(\sin v)+z_{i}(\cos v) \end{array}\right]=\left[\begin{array}{c} x_{f}\\ y_{f}\\ z_{f} \end{array}\right]\to \left[\begin{array}{ccc} 1 & 0 & 0\\ 0 & \cos v & -\sin v\\ 0 & \sin v & \cos v \end{array}\right]\left[\begin{array}{l} x_{i}\\ y_{i}\\ z_{i} \end{array}\right]=\left[\begin{array}{l} x_{f}\\ y_{f}\\ z_{f} \end{array}\right]$$

(F. XII) $$\to \left[M_{v}\right]\left[\begin{array}{l} x_{i}\\ y_{i}\\ z_{i} \end{array}\right]=\left[\begin{array}{l} x_{f}\\ y_{f}\\ z_{f} \end{array}\right]$$

And equations F. IX and F. X:


F. XIII $$\begin{array}{rl} & \left[\begin{array}{c} x_{i}(\cos t)-y_{i}(0)+z_{i}(\sin t)\\ x_{i}(0)+y_{i}(1)+z_{i}(0)\\ x_{i}(-\sin t)+y_{i}(0+z_{i}(\cos t) \end{array}\right]=\left[\begin{array}{c} x_{f}\\ y_{f}\\ z_{f} \end{array}\right]\to \left[\begin{array}{ccc} \cos t & 0 & \sin t\\ 0 & 1 & 0\\ -\sin t & 0 & \cos t \end{array}\right]\left[\begin{array}{c} x_{i}\\ y_{i}\\ z_{i} \end{array}\right]=\left[\begin{array}{c} x_{f}\\ y_{f}\\ z_{f} \end{array}\right]\\ & \to \left[M_{t}\right]\left[\begin{array}{c} x_{i}\\ y_{i}\\ z_{i} \end{array}\right]=\left[\begin{array}{c} x_{f}\\ y_{f}\\ z_{f} \end{array}\right] \end{array}$$

Matrices (M_h_) , (M_v_) and, or (M_t_) may be multiplied to produce different results. The products of matrices *are not commutative*, so the *order* in which they are multiplied *alters* the results. For matrices (M_h_) and (M_v_), one may have:


(F. XIV) $$\left(M_{h}\right)\left(M_{v}\right)=\left[\begin{array}{lcc} \cos h & \left(-\sin h)(\cos v\right) & \left(\sin h)(\sin v\right)\\ \sin h & \left(\cos h)(\cos v\right) & \left(-\cos h)(\sin v\right)\\ 0 & \sin v & \cos v \end{array}\right]$$

(F. XV) $$\left(M_{v}\right)\left(M_{h}\right)=\left[\begin{array}{ccc} \cos h & -\sin h & 0\\ \left(\sin h)(\cos v\right) & \left(\cos h)(\cos v\right) & -\sin v\\ \left(\sin h)(\sin v\right) & \left(\cos h)(\sin v\right) & \cos v \end{array}\right]$$

Therefore, when two different rotations are considered (for example of 30° and 40°) it is essential to know which of them (h or v) is = 30°. Of course, h = 30° (so v= 40°) is absolutely different from v = 30° (so h = 40°).

For example, for point P (x, y, z), the coordinates are named L (1, 0, 0) for x, A (0, 1, 0) for y and as S (0, 0, 1) for z, so P (L, A, S):


(F. XVI) $$\left(M_{h}\right)\left(M_{v}\right)\left(M_{p}\right)=\left[\begin{array}{ccc} L\cdot \cos h & -A\cdot (\sin h)(\cos v) & S\cdot (\sin h)(\sin v)\\ L\cdot \sin h & A\cdot (\cos h)(\cos v) & -S\cdot (\cos h)(\sin v)\\ 0 & A\cdot \sin v & S\cdot \cos v \end{array}\right]$$

**Figure 10:**
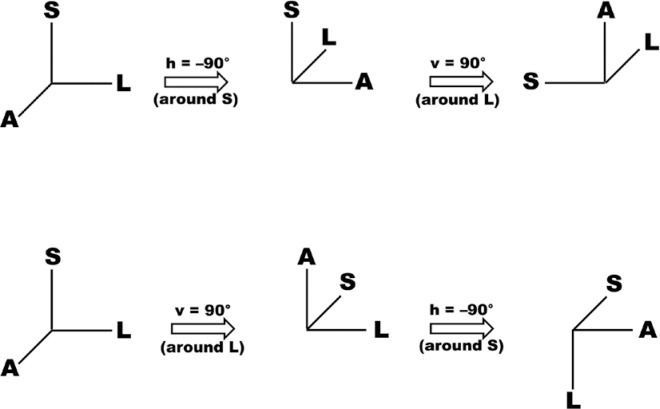
Schematic representation of differences in horizontal (h, around the ocular vertical axis) and vertical (v, around the ocular transversal axis) rotations of 90°, produced by changing the order which they are considered.

For cases where h = -90° (abduction) and v = 0° , x_f_ = A, y_f_ = -L, z_f_ = S. For h = 0° and v = 90°, x_f_ = L, y_f_ = -S , z_f_ = A. And for h = -90° and v = 90, x_f_ = -S, y_f_ = -L, and z_f_ = A. The initial and the final positions of points L, A, and S are visualized in Figure 10 (above).

However, for the product of matrices (M_v_).(M_h_) the results are quite different. Using the same set of coordinates for point P (L, A, S):


(F. XVII) $$\left(M_{v}\right)\left(M_{h}\right)\left(M_{p}\right)=\left[\begin{array}{ccc} L\cdot \cos h & -A\cdot \sin h & 0\\ L(\sin h)(\cos v) & A\cdot (\cos h)(\cos v) & -S\cdot \sin v\\ L\cdot (\sin h)(\sin v) & A\cdot (\cos h)(\sin v) & S\cdot \cos v \end{array}\right]$$

Therefore, for h = -90° and v = 0°, x_f_ = A, y_f_ = -L , z_f_ = S. For h = 0° and v = 90°, x_f_ = L, y_f_ = -S, z_f_ = A. For v = 90° and h = -90°, x_f_ = A , y_f_ = -S, z_f_ = -L (Figure 10, below).

Obviously, after a pure horizontal rotation, that is, if h = -90° (v = 0°), the final positions reached by the ocular axes are the same, independent of the product of matrices (F.XVI or F.XVII) used, that is x_f_ = A, y_f_ = - L, z_f_ = S (Figure 10, above). Similarly, after a *pure* vertical rotation, that is, if v = 90° (h = 0°), the final positions of the ocular coordinates for A, L, and S are the same, independent of the product of matrices (F. XVI, or F. XVII) used, that is, x_f_ = L, y_f_ = -S, z_f_ = A (Figure 10, below). But even when the values of angular coordinates are the same (h = -90°, v = 90°), *different* results emerge, according to the multiplication of matrices that is used (F.XVI *or* F.XVII), as Figure 10 shows. Of course, this could already be anticipated based on Figure 5. If one utilizes the angle measurements from the Fick system, point A (with the vertical rotation from B to F “ calculated after” the horizontal rotation from A to B, both around C) reaches the original position of S (Figure 10, above). If one uses the Helmholtz system, point A (with the horizontal rotation from D to G “calculated after” the vertical rotation, from A to D, both around C) reaches the original position of L (Figure 10, below). In fact, ocular rotations are *always* around C (or very close to an ideally fixed point in space) so it is merely due to the conventions for the expression of the angle coordinates that they may be taken to occur around different points (e.g., Z for F and X for G, Figure 5).

As a result of the several modes with which one may consider the angle coordinates used to measure an eye rotation on a plane (horizontal, sagittal or frontal): whether they occur at the center of ocular rotations and around movable (ocular) axes (*h, v,* and *t,* respectively) or around points on a fixed (orbital) referential axis (*H, V,* and *T,* respectively); and the order in which they are calculated, there are 48 possible combinations of these conventions (Table 1). However, the representation of eye positions by a system of polar coordinates does not preclude the knowledge of the order in which horizontal (h) and vertical (v) rotations are measured.

**Table 1:** Conventions for the measurement of the angular coordinates of horizontal (H or h), vertical (V or v) and torsional (T or t) rotations, according to whether they are taken to occur around the points of fixed (orbital) axes (vertical for H, transversal for V, and longitudinal for T) or around a fixed center of ocular rotations with movable (ocular) axes (for h, v and t), and the “order” in which the measurements are taken.

HVT	HTV	VHT	VTH	THV	TVH
HVt	HtV	VHt	VtH	tHV	tVH
HvT	HTv	vHT	vTH	THv	TvH
hVT	hTV	VhT	VTh	ThV	TVh
hvT	hTv	vhT	vTh	Thv	Tvh
hVt	htV	Vht	Vth	thV	tVh
Hvt	Htv	vHt	vtH	tHv	tvH
hvt	htv	vht	vth	thv	tvh

The need for a specific order of calculation had already been posited when coordinate systems for eye rotation measurements were first proposed^(^4^)^. Surprisingly, there remains no established and agreed-upon convention that has been shown preferable among the possible approaches to the measurement of ocular rotations. Since such rotations occur around a known center, it seems logical to select one of the six arrangements of measurements *h, v,* and *t*, shown in the last row of table 1.

While horizontal and vertical rotations are volitional and can be of relatively large magnitudes, torsional rotations are small and not subject to voluntary control. This lack of hierarchical and teleological importance (torsional rotations do not expand the oculomotor fields) suggests that in the measurement of ocular rotations, torsions (*t*) should be calculated *last.* If a choice must be made among the alternatives in the last row of table 1, then “hvt” or “vht” should prevail. Strictly, however, these cannot be taken as the representations of the Fick (Hvt) or Helmholtz (Vht) systems(*).

## RETROSPECTIVE SYNTHESIS

Finalities of vision (indirect contact with, and exploration of space) are essentially dependent on eye motions.

The complex functionality of **vision** is almost entirely dependent on movements. The delicate and *motionless* assembly of structures (retina) for capturing stimulation from the environment (light) to transform it in a mentally recorded “picture” benefits of *statically* concerted (“centered”) optical elements. But if such an ideally rigid construction (eye) were absolutely *immovable* the result would be of a limited image. Bodies could be seen traversing this framed space, though not being followed.

**Eye movements** are of two types: *translations*, when the eye as a whole - e.g., a “frozen” (immovable) eye - is spatially displaced by head movements; and *rotations*, when only one ocular point (its center of rotation) remain fixed, while all others are displaced relatively to the respective container (orbit). Although both of them are very important for the visual exploration of space, in clinical practice is usual to consider ocular movements as synonymous of ocular *rotations*.

**Ocular rotations** may occur in *any* direction of space, in a specific plane and around an imaginary axis perpendicular to it. They are formally defined according to the three, mutually orthogonal, spatial axes: one vertical (perpendicular to the horizontal plane and around which are defined the *horizontal* rotations) and two other horizontal axes, one perpendicular to the sagittal plane (the transversal ocular axis, around which are defined the *vertical* rotations) and another perpendicular to the frontal plane (the longitudinal ocular axis, around which are defined the *torsional* rotations). Exploration of the space is largely dependent on horizontal and vertical rotations which, therefore, may be voluntarily driven, while torsions do not contribute to enlarge the “visual fields”, so that usually small and reflexes to complement the former, volitionally commanded, eye rotations. Measurements of eye rotations may refer either to the *ocular* (movable) or to the *orbital* (fixed) system of coordinates.

The **primary position of gaze** is the landmark from where the measurements of eye positions and, or ocular rotations are made. This is conventionally defined as the (spatial) “placement” of the visual axis, the longitudinal (anterior to posterior) ocular axis, when the three *respective* axes of each (ocular and orbital) reference systems coincide. Note that “gaze straight ahead” - a construction frequently used to define the “primary position of gaze” - does not fulfill, necessarily, such a conception. Although “gaze” refers to “vision” (that is, the “position” or “direction” of the *ocular* longitudinal axis), while “straight ahead” address to “head” (that is, perpendicularly to the *orbital* frontal plane), that means that the ocular and the orbital longitudinal axes are coincident (gaze straight ahead), but the other two ocular axes (vertical and transversal) may be rotated (with torsion) relatively to the respective orbital axes.

**Measurements of ocular rotations** may be considered accordingly the two (ocular or orbital) reference system of coordinates. Except for the ocular poles at the primary position of gaze, *any* other point of the ocular surface is represented by different sets of coordinates. For instance (follow figure 5), for measurements of angular *horizontal* coordinates, the position of a specific point (E) may be expressed relatively to the vertical (orbital, fixed) reference system by an angle of “longitude” mea-sured from a standard meridian (AS, that of the orbital sagittal plane) on an arc of “small” circle (D’Z’E) of its “latitude”, centered at point Z’ of the vertical (orbital, fixed) axis of reference. But while this measurement may be labelled as “H” (equal to ACB’) a different angle measurement (‘h”), taken from the center of ocular rotation (C), may be also defined for the same point (angle DCE = angle ACB. All the same, for measurements of angular *vertical* coordinates, the same point E may be defined either by a value “V” (angle B’X’E = ACD’) or by a value “v” (angle BCE = ACD). Therefore, if E may be defined by two different horizontal angular coordinates (H or h, where h > H) and, or by two different vertical angular coordinates (V or v, where v > V), it may have *four* different combinations of vertical and horizontal coordinates (HV, Hv, hV and hv). If torsion is also considered, the same point have two different torsional mea-surements, accordingly the reference system elected (T or t), so that *eight* different set of coordinates result for defining the same point (HVT, HVt, HvT, Hvt, hVT, hVt, hvT, hvt). Hence, if the same point may be defined by different angular coordinates, conversely, the same set of coordinates may correspond to different points.

Besides the two (orbital, and ocular) system of coordinates with which the position of a point on the ocular surface (or of its projection) and, or of its trajectory may be defined, the *order* with which the rotations may be considered bring possible *six* possible arrangements (e.g., *hvt, htv, vht, vth, thv, tvh*). In fact, this corresponds to the *order* of how the correspondent matrix equations of each rotation in a plane are multiplied. As, for each arrangement (order) two different systems of coordinates are possible for defining each rotation, 48 alternatives result.

## OPERATION OF MEASUREMENTS

### The orthogonal superimposition of horizontal and vertical prims

The choice of a system of spherical coordinates and angle measurements has an important implication. In clinical practice, angle measurements of eye deviations (strabismus) are made using prisms. It is well known that such measurements can vary greatly depending on the position relatively to the eye that incident and emergent faces are. Therefore, specific rules must be followed when making these prismatic measurements. The preferred rule is probably the placement of the prism in a position, such that the face from which the light emerges coincides with the frontal plane of the respective orbit. (References to the positions on a prism “base”, as “nasal”, “temporal”, “superior” or “inferior” are merely conceptual and not technically valid. In fact, the meaning of “base” is simply of a “side”it does not imply a “face”. Besides, if a plane face exists as a “base”, it will not be a guide for the prism placement before the eye.) The greater the angle to be measured, the greater the apical angle of the prism. If a combination of horizontal and vertical measurements is necessary, an orthogonal superimposition of prisms can be used.

**Figure 11:**
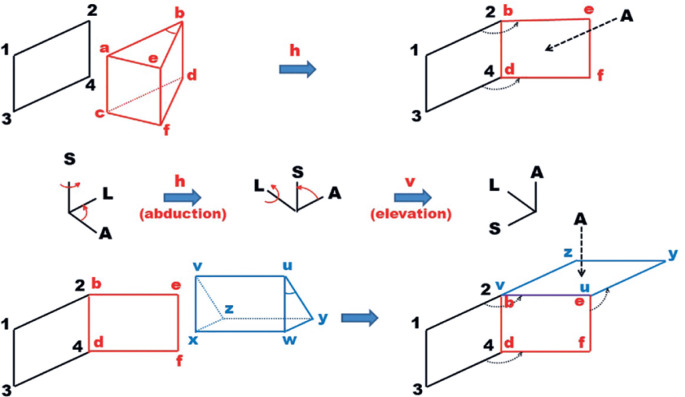
Above: A pictorial representation of the frontal plane of the left orbit (1234), with a prism (in red) for the measurement of an “outward” deviation. If the prism’s apical angle reaches 90°, the face that the light reaches (ebdf) becomes perpendicular to side 2-4, so that incident light comes from the *lateral* side. Below: A prism (in blue) to measure an “upward” deviation must be placed with its emergent face (vuxw) over face bedf. If the prism’s apical angle reaches 90°, the face that the light reaches (vuzy) becomes perpendicular to side b-e, so that incident light *comes from above* (compare with figure 12).

**Figure 12:**
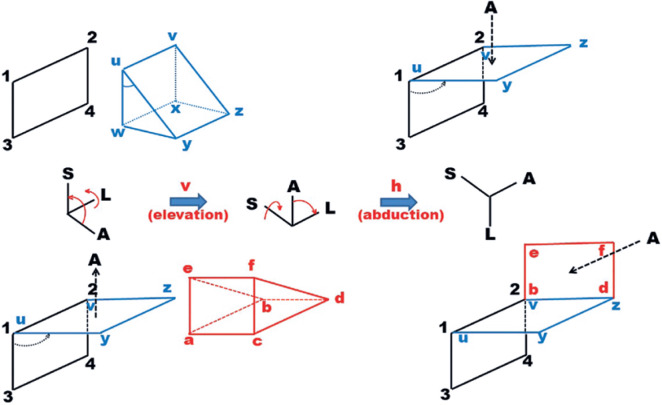
Above: A pictorial representation of the frontal plane of the left orbit (1234), with a prism (in blue) for measuring an “upward” deviation. If the prism’s apical angle reaches 90°, the face that the light reaches (uvyz) becomes perpendicular to side 1-2, so that incident light comes from above. Below: A prism (in red) measuring an “outward” deviation must be placed with its emergent face (abcd) over face uvyz. If the prism’s apical angle reaches 90°, the face that the light reaches (efbd) becomes perpendicular to side v-z, so that the incident light *comes from the left* (compare with figure 11).

Although *the relative positions of the horizontal and vertical prisms* (i.e., which of them is placed coincident to the frontal plane of the orbit), are rarely considered, differences in this relationship do exist as shown in Figures 11 and 12. If the horizontal prism is used “first” to the eye, the arrangement corresponds to the “hv(t)” system; if the vertical prism is used “first” to the eye, it corresponds to the “vh(t)” system. Obviously, this means that the way an arrangement of a horizontal and a vertical prisms is made before the eye affects the calculated magnitudes of deviations. (Figures 11 and 12 use *in extremis* illustrations of theoretical cases of prisms with apical angles of 90°. They are not intended to suggest that these angles occur.)

#### The accuracy and the significance of measurements

The evaluation of ocular movements is limited by two *natural* conditions, one *anatomical* and one *mechanical.* The *anatomical* limitation is the size of the smallest photoreceptor field at the center of the fovea (2 µm) of a human eye when measured from the second nodal point of the eye (17.055 mm before it, according to the classical Gullstrand’s reference)^(^5^)^ and corresponds to angle (a) given by arctan a = 2.10^-6^ m / 17.055.10^-3^ m, hence a ≈0.0067° ≈ 24.2”. This is in fair agreement with the discriminative limit of the optical ocular system, given by the radius size of the first circle of diffraction (Airy’s disc)^(^6^)^: A = 1.22 λ / d = 1.22 . 550.10^-9^ / 6.10^-3^ ≈ 112.10^-6^ rad ≈ 23.1” where λ is the wavelength of yellow light (550 nm) and d is the diameter of a “normal” pupil. Hence, the distance between two points that can be optically distinguished from one another, is twice the value of angle A, about 48” or 0.8’. However, this is an *optical* condition, and ocular rotations are measured from their rotational center, closer to the fovea (about 12.2 mm) than the second nodal point of the optical system. So the *mechanical* limiting condition is that of angle “e” estimated by 17 mm / 12,2 mm = e / 48”, that is, e ≈ 67” ≈ 1.1’.

The eye does not rest immobile but rather, is continuously in motion due to very fine oscillatory movements. These are categorized as high-frequency tremors (which may reach 1’), slow drifts (about 5’), rapid “flicks” or saccades (up to 20’) and other irregular movements (up to 5’)^(^7^)^. Together these occur within a retinal area about 100 µm in diameter^(^7^)^ (which corresponds to about 0.47° or 28’ from the center of ocular rotation). This is in accord with experiments concerned with the *physiological* evocation of a minimal ocular response: displacements smaller than 15’ to 30’ were not capable of eliciting saccades^(^8^)^. It is commonly accepted that is difficult, if not impossible, even for an experienced ophthalmic clinician, to detect an ocular deviation of such an order of magnitude (0.5°) with the naked eye.

#### Scales and unities

Angles may be quantified as degrees of an arc (an arc of 1° is 1/360 of a circumference) but also as *radians.* An arc of 1 rad has a length equal to the radius of the circumference. Hence 360° = 2 Π radians, or 1 rad = 180°/Π ≈ 57.296° ≈ 3437.747’(*). For measurements of the order of strabismus deviations, the centesimal part of the radian (the *cent-rad*) has been proposed as the unit of measurement^(^9^)^.

**Figure 13:**
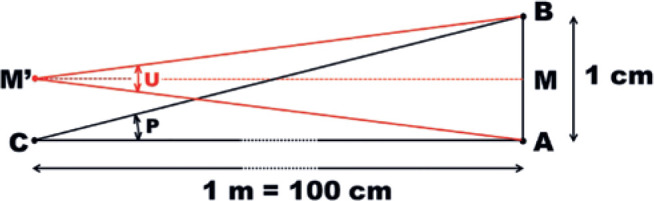
Prentice’s proposition for defining prism-diopter unity. Angle *P* corresponds to the 1 cm straight line (AB) perpendicularly taken at a distance (AC) of one meter (horizontal black line). If the same straight line lengths of 1 cm (AB) and 1 m (= MM’ = AC) are symmetrically placed (red lines), the new angular unity (u), although of almost the same absolute value (P = 0.999966669 c-rads, U=0,999991667 c-rads), provides greater mathematical conveniences.

However, the exact measurement of a curved line, such as the arc of a circle, presents practical difficulties. In 1890, Prentice proposed an estimative of angle measurements based on a simple relationship between two straight and perpendicular lines (Figure 13).^(^10^)^ This is calculated by determining the distance between two points (AB) and the distance between one of these points (say, A) and the point from which they are observed (C). The measurement unit, then defined as 100 AB/AC, was named *prism-diopter*. A small superscripted triangle is used to represent this unit (^Δ^). The benefits of such a simple measurement are evident. If one knows the distance between two points (say 2 m, which is the distance which separates a doubled image of an object) and the distance from which they are observed (say, 10 m), the ratio 100 x (2/10) = 20 represents the angle in prism-diopters (what is C - if the first principal point of the eye, the center of ocular rotation, or the fovea - has not been indicated? For clinical purposes, the possible differences related to distance AC are so small, relatively, as to be irrelevant).

The corresponding angle of 1^Δ^ = 1 cm/ 1 m = 0.01, that is, tan a = 0.01, leads to a ≈ 0.5729**38697**°. The value of this angle (a) is almost “exactly” the same as that of a cent-rad (1 cent-rad = 1.8 / Π ≈ 0.572957795° a difference of 0.003333 % ≈ 1/30000), which allows an acceptable estimate of 4° ≈ 6.98 cent-rad ≈ 7^∆^. The major problem is that while this direct (“linear”) approximation holds for relatively small angle values, it does not allow arithmetic operations with prism-diopter unities. For example, 2 x 40° = 80°. But 40° (≈83.91^Δ^) + 40° (≈ 83.91^Δ^) = 80° (≈ 567.13^Δ^). A related question concerns the superimposition of prisms, for which the analysis is still more complex. Two prisms with n = 1.49 and 40^Δ^, superimposed so their apices are coincident, evoke an angle deviation of 337.87^Δ(^11^)^.

What is known as the practical rule of Prentice, i.e., using a ratio of straight lines (two orthogonal distances, *x* and *d*) to express angle measurements, is actually a *tangent* scale, since a measurement in prism-diopters (P) is P^Δ^ = 100 tan (x/d), which requires the rather disturbing conversion of a 90° angle to an *infinite* value in prism-diopters. Crescent angle values of 90° to 180° are converted to *decreasing* and *negative* values in prism-diopters. However, a new way of defining the same prism-diopter unity (Figure 13) changes such discrepancies enormously. An angle of 90° becomes 200 u and crescent angle values of 90° to 180° increase in a *positive* direction (up to an infinite value for the expression of 180°). Mathematically, the angle measurement (U) with the new unity is simply given by^(^12^)^:


(F. XVIII) U=200tan⁡(x/2d)

**Figure 14:**
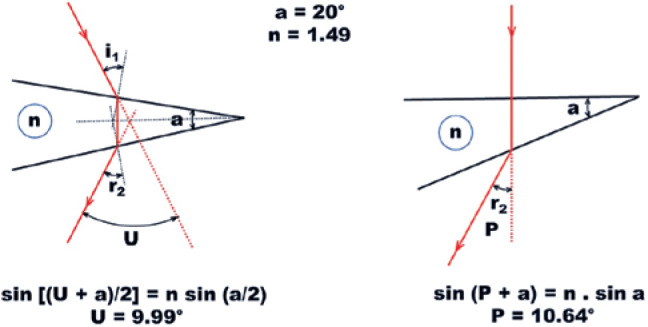
Condition of minimum deviation (U = 9.99°) produced by a prism with an apical angle (a) of 20° where n=1.49 (left) and the deviation (P) produced by an incidence of 0°, Prentice’s condition (right) is 10.64°.

An additional theoretical advantage of this convention is that it conforms to the existing practical convention used to define a prism angle value by evoking its *minimum deviation*. This *angle of minimum deviation* is precisely produced by the *symmetrical* positioning of the incident and refracted rays relative to the optical surfaces of the prism (Figure 14, left) equivalent to their symmetrical positions relative to the object and the observation point. This is similar to the “split” position used to define the new angular unity (Figure 13).

If the new scale (U) is used with a prism previously found to be 50 cent-radians (≈28.648°) and 54.630 prism-diopters, the value in this new scale drops to 51.068 u. Still better approximations may be reached, if the “divide and multiply” criteria are used^(^13^)^. For instance, the value becomes 50.262 unities if one defines the unity in the new scale (U_4_) using the formula U_4_ = 400 tan (a/4), or 50.002 unities if the formula is U_50_ = 5000 tan (a/50).

The exact relationship between a ratio of two orthogonal distances (*x* and *d*), a tangent scale, which provides the basic principle for defining prism-diopter (P) or unities at this “split” scale (U), as an angle mea-surement in degrees of arc (or radians), may be perfectly reached using the equation


(F. XIX) $$U_{k}=(100k)\tan(a/k)$$

when k = ∞. If, for instance, k = 10, an angle (a) of 90° (= 157.080 cent-radians) can be converted to U_10_ = 1000 tan 9° = 158.384 unities, with an error of only 0.83% relative to the angle value in cent-radians. Table 2 shows angle values in degrees of arc, the corresponding conversions to cent-radians, the new angular unity values (U_k_) according to the chosen convention for its calculation (k) and the respective percent errors (e), where e = 100 (U_k_ - c) / c.

**Table 2:** Values of an angle in degrees of arc (a); cent-radians (c) (cent-radian = a . Π / 1.8) ; prism-diopters (P) (where P = tan a); the numeric value of a new angular unity (U_2_) = 200 tan (a/2); or greater approximations to a linear variation (U_10_) = 1000 tan (a/10); and its respective percent errors of the exact measurements. Still smaller errors are reached by the scale (U_100_) = 10000 tan (a/100).

a degrees	c centiradians	P Prism-diopt.	U_2_ New unity	U_1O_ New unity	U_1O0_ New unity	Error U_10_/c
1’	1.74533	1.74551	1.74537	1.74533	1.74533	0.00010%
45°	78.53982	100.00000	82.84271	78.70171	78.54143	0.20613%
90°	157.07963	00	200.00000	158.38444	157.09255	0.83067 %
135°	235.61945	-100.00000	482.84271	240.07876	235.66306	1.89259%
180°	314.15927	0	00	324.91970	314.26267	3.42515%
360°	628.31853	0	0	726.54253	629.14667	15.63283%

Even though an angle of 45° is too large to occur much in the measurement of eye rotations, and deviations, in clinical practice, the most basic “split” unity (U_2_, shown in figure 13), can measure it with an “absolute” error (e), where e = 100 (U_2_ - c) / c of only about 5% (i.e., 82.84271 / 78.53982 ≈ 1,0548).

## Footnotes

(*) : If measurements are begun at the primary position of gaze where, by definition, ocular (movable) and orbital (fixed) axes are coincident, then an “initial” rotation around a vertical (SI) axis may be taken as H or h; an “initial” rotation around a transversal (LM) axis may be taken as V or v; and an “initial” rotation around a longitudinal (AP) axis may be taken as T or t. This reduces the 48 arrangements shown in Table 1 to 24. In this case, the Fick’s system is represented by *Hvt* or *hvt*, while Helmholtz’s system is represented by *Vht* or *vht*.

(*): The elegant approximation of 22/7 (≈ 3.14286) for the value of Π (≈ 3.14159) proposed by Archimedes gives 1 rad ≈ (90 x 7 / 11) ≈ 57.2727…° an error of slightly more than 0.04%. But with the repetition of the first three odd numbers (113355) to obtain the ratio of a numerator (355) and denominator (113), so, with the fraction 355/113, one reaches an approximation of the value of Π with an error that does not reach 8.5x10^-6^ % !

## References

[1] Bicas HEA. (2009). Strabismus: from theory to practice, from concepts to its
operational attainment. Arq Bras Oftalmol.

[2] Park RS, Park GE. (1933). The center of ocular rotation in the horizontal
plane. Am J Physiol.

[3] Fry GA, Hill WW. (1962). The center of rotation of the eye. Am J Optom.

[4] Bicas HEA (2006). Proceedings of the Joint Congress The Xth Meeting of the International
Strabismological Association and the First Extraordinary Meeting of the
Latinamerican Council of Strabismus.

[5] Gullstrand A., Southall JPC (1924). Helmholtz’s Treatise on Physiological Optics.

[6] Fincham WHA. (1965). Optics.

[7] Alpern M., Davson H (1969). Types of movement. In: The eye.

[8] Rashbass C. (1961). The relationship between saccadic and smooth tracking eye
movements. J Physiol.

[9] Dennet WS. (1889). A new method of numbering prisms. Tr Am Ophthalmol Soc.

[10] Prentice CF. (1890). A metric system of numbering and measuring prisms. Arch Ophthalmol.

[11] Bicas HEA. (1980). Efeitos rotacionais mono e binoculares das
associações de prismas. Rev Bras Oftalmol.

[12] Bicas HEA., Bicas HEA, Souza-Dias C, Almeida H (2007). Estrabismos.

[13] Bicas HEA. (2014). A new unity for angular measurements in
strabismus. Arq Bras Oftalmol.

